# SCPNB is an adjuvant to local anaesthesia for maxillofacial surgical practice

**DOI:** 10.6026/97320630019605

**Published:** 2023-05-31

**Authors:** Sachin Kumar, Pranav Parashar, Saurabh Mallick, Dr. Abhishek, Sanjivani L. Mane, Adsure Girija

**Affiliations:** 1 Department of Oral and Maxillofacial Surgery School of Dental Sciences, Sharda University, Greater Noida, UP, India; 2 Department of Dentistry, N.S.C.B. Medical College & Hospital, Jabalpur, M.P, India; 3 Department of Oral & Maxillofacial Surgery, Mallick Dental & Maxillofacial Centre, Ranchi, Jharkhand, India; 4 Department of Oral and Maxillofacial Surgery Oro Care Facial Trauma Centre, Patna, Bihar, India; 5 Department of General Pathology and Microbiology, MA Rangoonwala College of Dental Sciences & Research Centre, Pune, Maharashtra, India; 6 Department of Orthodontics & Dentofacial Orthopaedics, MA Rangoonwala College of Dental Sciences & Research Centre, Pune, Maharashtra, India

**Keywords:** Superficial cervical plexus nerve block, alternative, local anaesthesia, maxillofacial surgery

## Abstract

The superficial cervical plexus nerve block [SCPNB] procedure is frequently used throughout head and neck surgery because it is simple to learn and has a low rate of complications. The investigation of this method might produce superior outcomes in treating frequent maxillofacial disorders including mandibular fractures and infections of the odontogenic region. The SCPNB is known to play a part in the medical evacuation of head and neck abscesses, the excision of superficial diseases in the perimandibular region, and the therapy of mandibular fracture, despite the dearth of research in this area. Considering this background, it was expected that the SCPNB might be helpful as an adjuvant to regional anaesthesia in maxillofacial surgery. The purpose of this study was to assess the effectiveness of the SCPNB in the treatment of mandibular fractures and infectious diseases in the perimandibular area. 48 patients with either submandibular space infections or mandibular injuries who were anticipated for surgical procedure under regional anaesthesia participated in a prospective randomized clinical study (eg, inferior alveolar nerve block, long buccal nerve block). Administering a combination of a local infiltration and regional anaesthesia was used as the control group. Regional anaesthesia and a SCPNB were administered to the intervention class. The following factors were examined: pain, anaesthesia's duration and onset, waiting period before initial analgesic demand, pulse rate, and blood pressure. The unpaired t-test was used to compare groups. Multiple variables ANOVA (for more than two observations) was used for intragroup analysis, accompanied by a post-hoc analysis of variance. In aspects of intra - operative pain at thirty minutes, time required of anaesthesia, intraoperative anesthetic necessity, duration until first analgesic recommendation, and intra - operative diastolic arterial blood pressure at ten minutes, the SCPNB group demonstrated a substantial (P ≤.01) improved performance. It can be concluded that the use of a regional anaesthetic approach in conjunction with a SCPNB is a good substitute to localized infiltration for patients having surgery for fracture of mandible and perimandibular area infections.

## Background:

 Dental procedures and oral surgical treatments are frequently carried out in outpatient facilities. The most popular way to anaesthetize a patient before office-based operations is regional anaesthesia. The upper jaw, lower jaw along with the tissues, both soft and hard, can all be anaesthetized using a variety of procedures. The technique of anaesthesia to be utilized will depend on the kind of treatment to be done as well as the region of the surgery. Despite being straightforward and uncomplicated to conduct, the superficial cervical plexus block popularly considered as SCPB is regrettably frequently disregarded as a substitute for general anaesthesia (GA) [1]. In modern medicine (GA) is a relatively safe, practical, and easy method to achieve surgical anaesthesia. The drawbacks of GA include its high financial expense, the need for numerous highly skilled individuals, mortality and morbidity, and expensive equipment. The benefits of regional anaesthesia encompass a reduced amount of hemorrhage due to local vascular constrictors and sympathetic suppression, stress-free anaesthesia because it hinders excessive catecholamine discharge, simple method of administration, and lesser morbidity incidences with acceptable quantities of local anaesthesia (LA) [2]. Halstead administered the first cervical plexus block (CPB) at Bellevue, New York, in 1884. Subsequently, Kappis documented the posterior approach in Germany. Although Heidenhein invented the lateral strategy, Labat is credited with making it widely used in United States. [3, 4] The SCP blockade has been extensively described for anaesthesia of the neck region, submandibular region, and lobes of the ears and may be helpful for patients who have wounds on their ear lobes, boils in the submandibular region, or neck traumas. Its use in the field of oral and maxillofacial surgery (OMFS) has included the stitching of skin in the appropriate dermatome, removal of superficial wounds, and surgical evacuation of an abscess in the perimandibular zone. [5,6] The superficial cervical plexus nerve block [SCPNB] procedure is frequently used throughout head and neck surgery because it is simple to learn and has a low rate of complications. The investigation of this method might produce superior outcomes in treating frequent maxillofacial disorders including mandibular fractures and infections of the odontogenic region. The SCPNB is known to play a part in the medical evacuation of head and neck abscesses, the excision of superficial diseases in the perimandibular region, and the therapy of mandibular fracture, despite the dearth of research in this area. [7,8] Therefore, it is of interest to evaluate as an adjuvant to regional anaesthesia in maxillofacial surgery.

##  Material and Methods:

48 patients with either submandibular space infections or mandibular injuries who were anticipated for surgical procedure under regional anaesthesia participated in a prospective randomized clinical study (eg, inferior alveolar nerve block, long buccal nerve block). Administering a combination of a local infiltration and regional anaesthesia was used as the control group. Regional anaesthesia and a SCPNB were administered to the intervention class. The following factors were examined: pain, anaesthesia's duration and onset, waiting period before initial analgesic demand, pulse rate, and blood pressure at time duration of 10 minutes, 15 minutes, 30 minutes, 60 minutes, 120 minutes. The study participants were divided into two categories

Category A: Combination of a local infiltration and regional anaesthesia (n=25)

Category B: Regional anaesthesia and a SCPNB. (n=23)

## Criteria for inclusion

Healthy individuals among the ages of 15 years and 60 years who required surgical treatment in lower jaw area and perimandibular regions along the course of the superficial cervical plexus met the requirements for inclusion, as did individuals who were prepared to engage in the study following careful consideration and an in-depth description of the procedure.

## Criteria for exclusion

Participants with medical problems, individuals who are overly fearful and worried those who don't want the treatment to be conducted under regional anaesthesia, and those who have a history of local anaesthetic allergies were all excluded from the study. Simultaneously with the additional nerve blocks, all of the patients received a SCPB in category B. The similar maxillofacial specialist administered SCPB after thoroughly examining the regional anatomy and under constant anaesthesia surveillance by the anesthesiologist in a properly furnished operating room.

## Armamentarium used

[1] A 4-5 cm, short bevel, 22-gauge needle.

[2] 10-15 millilitres of local anaesthetic.

[3] Marker.

[4] Ideally a vitals monitoring device.

## Procedure:

All aseptic safeguards were taken when preparing and cleaning the patient. The patient was lying on their back with a tiny towel under their head, which was tilted slightly towards the opposite direction that has been anaesthetized, and their vital signs were continuously monitored by the anesthetist throughout the treatment. The person receiving anaesthesia was told to elevate their head while being gently resisted by the anesthetist's forearm. To aid identify the exterior jugular vein and delineate the sternocleidomastoid muscle, a modest Valsalva's manoeuvre was recommended at the same time. The central location of the sternocleidomastoid muscle's posterior margin was identified and highlighted. This typically lines up with the exterior jugular vein when it enters the muscle. In order to infiltrate 5-10 mL of local anaesthetic, a 22-gauge, 4-cm needle was inserted 2-3 cm in superior direction and inferior direction into the subfascia along the muscle's boundary. There was no search for paresthesia. After the local anaesthetic was injected, ten to fifteen minutes were given before the effectiveness of anaesthesia was evaluated. Following the anaesthesia along the SCP's dispersion, objective symptoms were examined, and the surgical process was completed. The identical surgeon executed all of the participants' surgical operations under regional anaesthesia (SCP block with additional nerve blocks) with effective anaesthesia and analgesia and no complications. Along with the additional nerve blocks, all of the patients received a SCPB. The same maxillofacial operator administered SCPB after thoroughly examining the regional anatomy and under constant anaesthesia surveillance by the anesthesiologist in a properly furnished operating room.

## Statistical analysis:

SPSS version 2021 was used for carrying out the statistical analysis. The unpaired t-test was used to compare groups. Multiple variables ANOVA (for more than two observations) were used for intragroup analysis, accompanied by a post-hoc analysis of variance. The findings were considered statistically viable when the p value was less than or equal to 0.05.

## Result

The demographic details of study participants of category A and category B has been documented in Table 1. The mean age of study participants in category A was 31.23±1.4 years while the mean age of study participants in category B was 32.45±1.1 years. Males constituted 67.34% of total study participants in category A while males constituted 68.21 of total study participants in category B. 16 (33.34%) study participants in category A and 17 (35.41%) study participants in category B were diagnosed with submandibular space infections. 17 (35.41%) study participants in category A and 16 (33.34%) study participants in category B were diagnosed with maxillofacial injuries. 8 (16.67) study participants in category A and 7 (14.58) study participants in category B were diagnosed with dentigerous cyst or radicular cyst. 7 (14.58) study participants in category A and 8(16.67) study participants in category B were diagnosed with submandibular space infections. Incision and drainage was carried out in 68.75% study participants in both category A and category B. Open reduction and internal fixation was carried out in 35.41% study participants in category A and 33.34% study participants in category B. Enucleation was carried out in 16.67% study participants in category A and 14.58% study participants in category B. Among complications, the post-operative swelling was observed in 4.17% study participants in category A, while post-operative swelling was observed in 2.09% study participants in category B. The post-operative paresthesia was observed in 2.09% study participants in category A, while post-operative paresthesia was observed in 4.17 % study participants in category B. The difference in demographic details among study participants in category A and category B was non-viable statistically (p> 0.05) as shown in Table 1.

The VAS scores in both categories has been documented in Table 2. It was observed that difference in intraoperative VAS level at 15, minutes and 30 minutes in both categories was significant statistically (p≤ 0.05). The pain scores was lower in study participants of category B. However the pain level at 60 minutes and 120 minutes time interval was similar in both category A as well as category B. ( p> 0.01).(Table 2).

The waiting period before initial analgesic demand in study participants after completion of surgery in category A was 75.12±14.24 min. The waiting period before initial analgesic demand in study participants after completion of surgery in category B was 137.23 ± 32.43 min. The difference was viable statistically (p ≤ 0.001) with study participants in category A asking for analgesics earlier than that of study participants of category B. (Table 3

The information regarding onset and duration of anaesthesia has been documented in Table 4. The mean onset duration was 98.23 sec in study participants of category A. The mean onset duration was 87.12 sec in study participants of category B. The difference was viable statistically.(p=0.03). The mean duration of anaesthesia in study participants in category A was 3.21 hours while it was 4.24 hours in study participants in category B. The difference was viable statistically. (p=0.02). The onset of anaesthesia was earlier in category A and duration of anaesthesia was greater in study participants of category B. (Table 4).

It was observed that pulse rate at different time intervals in study participants of category A and category B was comparable and the difference was not significant statistically (p≥0.05) (Table 5). On the other hand the diastolic arterial pressure was in more stable among study participants in category B at 10 minute intra-operatively. The difference was significant statistically. (p≤ 0.05). (Table 6)

In aspects of intra - operative pain at thirty minutes, time required of anaesthesia, intraoperative anaesthetic necessity, duration until first analgesic recommendation, and intra - operative diastolic arterial blood pressure at ten minutes, the SCPNB group demonstrated a substantial (P ≤.01) improved performance.

## Discussion:

Anaesthesia and pain relief are provided to the head and neck area by the cervical plexus block. Pain control is a crucial part of maxillofacial surgery. Traditional local anaesthetic blocks provide sufficient anaesthesia but are insufficient in some circumstances, such as perimandibular space illnesses where an abscess extends into the more profound facial spaces, submandibular lesions along with cervical lesions that need to be dissected in the deeper planes, and fracture of angle of mandible. General anaesthesia is frequently used in these circumstances, although it has drawbacks including costly expenses, morbidity, and mortality. Surgery may be performed safely and comfortably on patients in deeper areas of the neck area including perimandibular area thanks to SCPB's efficient use of LA. [9,10] In this study the VAS scores in both categories has been documented in Table 2. It was observed that difference in intraoperative VAS level at 15, minutes and 30 minutes in both categories was significant statistically (p≤ 0.05). The pain scores was lower in study participants of category B. However the pain level at 60 minutes and 120 minutes time interval was similar in both category A as well as category B. It showed that pain was lesser on administration of regional anaesthesia and a SCPNB.

In this research the waiting period before initial analgesic demand in study participants after completion of surgery in category A was 75.12±14.24 min while the waiting period before initial analgesic demand in study participants after completion of surgery in category B was 137.23 ± 32.43 min. The difference was viable statistically (p ≤ 0.001) with study participants in category A asking for analgesics earlier than that of study participants of category B. It showed that waiting period before initial analgesic demand was greater after administration of regional anaesthesia and a SCPNB.

According to Kamal Kanthan's [11] observation in a study involving 10 patients, excellent anaesthesia and successful outcomes were achieved by combining SCP block with standard nerve blocks like the inferior alveolar nerve blocks and long buccal nerve blocks. Arun [12] employed this block to treat Ludwig's angina and came to the conclusion that it was a viable alternative for a remote hospital with scarce assets because it allowed for decompression via surgery in their circumstance. To get optimal clinical results, one needs to have a detailed understanding of the relevant anatomy and the correct block technique.

In this study the mean age of study participants in category A was 31.23±1.4 years while the mean age of study participants in category B was 32.45±1.1 years. Males constituted 67.34% of total study participants in category A while males constituted 68.21 of total study participants in category B. 16 (33.34%) study participants in category A and 17 (35.41%) study participants in category B were diagnosed with submandibular space infections. 17 (35.41%) study participants in category A and 16 (33.34%) study participants in category B were diagnosed with maxillofacial injuries. 8 (16.67) study participants in category A and 7 (14.58) study participants in category B were diagnosed with dentigerous cyst or radicular cyst. 7 (14.58) study participants in category A and 8(16.67) study participants in category B were diagnosed with submandibular space infections.

In our research incision and drainage was carried out in 68.75% study participants in both category A and category B. Open reduction and internal fixation was carried out in 35.41% study participants in category A and 33.34% study participants in category B. Enucleation was carried out in 16.67% study participants in category A and 14.58% study participants in category B. Among complications, the post-operative swelling was observed in 4.17% study participants in category A, while post-operative swelling was observed in 2.09% study participants in category B. The post-operative paresthesia was observed in 2.09% study participants in category A, while post-operative paresthesia was observed in 4.17 % study participants in category B. The difference in demographic details among study participants in category A and category B was non-viable statistically. (p> 0.05).

In outpatient clinics, dental procedures and oral surgical treatments are often performed. Regional anaesthetic is the most frequently used technique to put a patient to sleep before office-based procedures. There are numerous ways to anaesthetize the soft and hard tissues, as well as the upper and lower jaws. The type of procedure to be performed as well as the site of the surgery will determine the anaesthetic technique to be used. The superficial cervical plexus block, often known as SCPB, is regretfully frequently neglected as a replacement for general anaesthesia (GA) while being simple and easy to perform [13-14,15].

In contemporary medicine, achieving surgical anaesthetic can be done easily, safely, and practically with (GA). GA has a number of disadvantages, including high costs, the requirement for numerous highly experienced workers, mortality and morbidity rates, and expensive equipment.[1,13,14] The advantages of regional anaesthesia include less bleeding because of local vascular constrictors and sympathetic suppression, stress-free anaesthesia because it prevents excessive catecholamine release, a straightforward administration method, and lower rates of morbidity with appropriate levels of local anaesthesia (LA). [16-17, 18] Sickness, bleeding phrenic nerve obstruction, LA toxic effects, nerve injury, and spinal anaesthesia are just a few of the potential side effects and repercussions of CPB, albeit if they are properly managed, they frequently have little to no effect [19-20,21].

The absence of any instances of harmful drug or technique use in a prior case series is consistent with preceding research. Accidental deep injections of local anaesthesia, which block deeper neurological networks such the recurrent laryngeal nerve, cervical plexus, and brachial plexus, are the most frequent side effects of the SCP block [122]. However, these issues are incredibly rare and are readily avoidable by adhering to the basic block security procedures. The mean onset duration was 98.23 sec in study participants of category A. The mean onset duration was 87.12 sec in study participants of category B. The difference was viable statistically (p=0.03). The mean duration of anaesthesia in study participants in category A was 3.21 hours while it was 4.24 hours in study participants in category B. The difference was viable statistically.(p=0.02). The onset of anaesthesia was earlier in category A and duration of anaesthesia was greater in study participants of category B.

It was observed that pulse rate at different time intervals in study participants of category B and category B was comparable and the difference was not significant statistically (p≥0.05) (Table 5). On the other hand the diastolic arterial pressure was in more stable among study participants in category B at 10 minute intra-operatively. The difference was significant statistically ( p≤ 0.05).

For patients with wounds on their ear lobes, boils in the submandibular region, or neck injuries, the SCP blockade has been extensively detailed for anaesthesia of the neck region, submandibular region, and lobes of the ears. It has been used in the field of oral and maxillofacial surgery (OMFS) for the surgical evacuation of an abscess in the perimandibular region as well as the stitching of skin in the appropriate dermatome and the treatment of superficial wounds. [23] Because it is easy to learn and has a low rate of problems, the superficial cervical plexus nerve block [SCPNB] method is routinely utilised throughout head and neck surgery. The examination of this technique may result in better treatment outcomes for common maxillofacial conditions such mandibular fractures and odontogenic area infections. Despite the paucity of research in this field, it is known that the SCPNB contributes to the treatment of mandibular fractures, the excision of superficial illnesses in the perimandibular region, and the medical evacuation of head and neck abscesses. [24- 25]

## Conclusion:

Data shows that the use of a regional anaesthetic approach in conjunction with a SCPNB is a good substitute to localized infiltration for patients having surgery for fracture of mandible and peri-mandibular area infections.

## Figures and Tables

**Table 1:** Demographic details

	**Category A**	**Category B**	**P value**
Age (mean ± SD) years	31.23±1.4	32.45±1.1	0.13
Male (%)	67.34	68.21	0.47
Diagnosis
Submandibular space infections, n (%)	16 ( 33.34)	17 ( 35.41)	0.12
Mandibular injuries, n (%)	17 (35.41)	16 (33.34)	0.11
Dentigerous cyst/ Radicular cyst, n (%)	8 (16.67)	7 (14.58)	0.27
Submandibular space infection , n (%)	7 (14.58)	8 (16.67)	0.34
Treatment procedures
Incision and drainage (%)	68.75	68.75	0.22
Open reduction and internal fixation (%)	35.41	33.34	0.14
Enucleation (%)	16.67	14.58	0.17
Complications
Post operative swelling (%)	4.17	2.09	0.56
Paresthesia (%)	2.09	4.17	0.72

**Table 2:** VAS score in both categories

	**Category A**	**Category B**	**P value**
10 min	4	4	0.781
15 min	4	3	0.002
30 min	3	2	0.001
60 min	2	2	0.891
120 min	1	1	0.762

**Table 3:** Waiting period before initial analgesic demand

	**Category A**	**Category B**	**P value**
When the patient will initially request analgesia following surgery (minutes)	75.12± 14.24 minutes	137.23 ± 32.43 minutes	0.0312

**Table 4:** Onset and duration of anaesthesia

	**Mean onset (sec)**	**Mean Duration (hr)**
Category A	98.23	3.21
Category B	87.12	4.24
P value	0.03	0.02

**Table 5:** Pulse rate in both categories

	**Category A**	**Category B**	**P value**
10 min	73.34	74.62	0.23
15 min	71.12	71.37	0.45
30 min	73.47	72.48	0.76
60 min	75.21	76.23	0.81
120 min	74.12	75.43	0.65

**Table 6:** Diastolic arterial pressure

	**Category A**	**Category B**	**P value**
10 min	93.12± 3.4	84.34± 1.2	0.014
15 min	92.34±2.2	80.25± 1.7	0.002
30 min	90.12± 4.3	87.34±2.1	0.003
60 min	89.34±1.2	84.23±3.4	0.004
120 min	91.21±1.4	83.23±1.4	0.002
